# The Camino de Santiago as a ‘Spiritual Journey’: A Contemporary Challenge to Religion?

**DOI:** 10.1007/s10943-024-02108-2

**Published:** 2024-08-29

**Authors:** Piotr Roszak, Franciszek Mróz

**Affiliations:** 1 Faculty of Theology, Nicolaus Copernicus University in Toruń Ul, Gagarina, 3787100 Toruń, Poland; 2 Department of Tourism and Regional Studiesul, Krakow Institute of Management and Social Affairs, University of the National Education Commission, Podchorążych, 230084 Kraków, Poland

**Keywords:** Depression, Flourishing, Meaning, Pilgrimage, Spirituality, Spiritual health, Well-being

## Abstract

According to many surveys, the pilgrimage along the Way of St James (Camino de. Santiago) can lead to spiritual benefits, but there is some disagreement about this because these benefits can be associated with the pilgrim's motivation. This article presents a conceptual framework for understanding the phenomenon of pilgrimages to Compostela and their impact on human spiritual well-being. Many diaries mention various positive psychological effects from these trips, but they are presented in religious/spiritual dialectical tension. The article presents the classical concept of spirituality as related to the ability to transcend, and then classifies what is spiritual in the writings of some Polish pilgrims. In this way, conceptual precision will be offered, which is important for understanding the positive impact of pilgrimages on well-being and empowerment.

## Introduction

The Camino de Santiago, a pilgrimage route leading to the Cathedral of St. James in Santiago de Compostela, is one of the most important and oldest pilgrimage routes in Christianity and also the first European Cultural Route (Cazaux, 2011; Mróz et al., 2019; Roszak, 2023a; Zhang et al., 2024). The last quarter-century has registered tremendous growth in both the Camino de Santiago network in Europe (more than 80,000 km of marked trail; Mróz, 2020) and pilgrimages and hikes along the route (Roszak, 2019).

In a number of works in the recent literature, researchers emphasise that pilgrims engage in seeking diverse experiences, improving psychophysical well-being, a more integrated spiritual life, establishing social ties, deepening knowledge (e.g., Collins-Kreiner, 2016; Klimiuk & Moriarty, 2021; Platovnjak & Zovko, 2023; Vistad et al., 2020). Researchers also expose the impact of pilgrimage on quality of life, the level of well-being of individuals and its implications for people's lives (Latusi & Fissore, 2021). The richness of experiences, encounters on the Way of St. James affects all spheres of the pilgrim-wanderer: intellectual, physical, emotional and spiritual. Pilgrimage is a special time for the realisation of values, conversation with God, learning about the world and oneself (wandering into the “depths of oneself”), a way of being with others on the road, and a unique time of mental and spiritual renewal (Feliu-Soler et al., 2021; Morris, 1982; Roszak, 2023b; Tykarski & Mróz, 2024). Our personal experiences and observations from multiple pilgrimages along the Way of St. James, as well as hundreds of conversations with pilgrims on the Camino de Santiago, lead us to conclude that the Way of St. James is a special space for encountering, seeking and finding, conversion, changing and appreciating life, strengthening self-acceptance and concern for others, abandoning superficial living, and realising human freedom (Brumec, 2022; Roszak, 2019; Seryczyńska, 2019; Zhang et al., 2024). The landscape of the Camino de Santiago promotes physical, emotional, spiritual, intellectual and social well-being and affects the senses of the pilgrim – traveller traversing this route (Faria et al., 2024).

Research by Harris and Wolf confirms that cardiovascular disease risk factors can be improved in healthy subjects participating in a low intensity, long duration, high frequency activity such as a walking pilgrimage on the Camino de Santiago (Harris et al., 2013). Harris emphasises that the physiological challenges of walking the Way of St. James have a strong impact on the pilgrim's overall experience, and for many pilgrims, the physical challenge of walking the trail is a significant psychological barrier to overcome (Harris, 2019). Pilgrimage – hiking together for married couples contributes to deepening marital relationships, getting to know each other better, strengthening bonds, enhancing trust, showing care and affection, and improving relationships with children (Tykarski & Mróz, 2024).

The development of new technologies in the field of social robotics also makes it possible to use teleoperated robotic avatars to “hike” the pilgrimage routes of people with disabilities, as exemplified by the “Santi” robot, a Unitree Go1 model from the University of Vigo, which became the “eyes” and “feet” of Elisa, a Cisco employee with limited mobility from Italy, who remotely guided the avatar on the last 90 m to the Cathedral of Santiago de Compostela (Hernández, 2022).

It is also necessary to emphasise that the pilgrimage route to Santiago de Compostela has been a test of endurance for pilgrims since its inception. The Camino de Santiago is also a space where many pilgrims have completed their earthly journey – this is evidenced by the many monuments erected by loved ones who lost their lives on the way to Compostela. Research conducted by Felkai (2019) confirms that the main cause of death during the pilgrimage on the Way of St. James is ACS (Acute Coronary Syndromes), followed by road accidents, and fatal exacerbation of a pilgrim's pre-existing illness and ailments caused by extreme weather conditions (Felkai, 2019).

Among the many perceptions of the Camino de Santiago, surveys and travel accounts note the phrase ‘spiritual journey’, which is sometimes understood as a counter-proposal to the religious focus of the pilgrimage (Margry, 2015; Ollivier, 2011; Silva et al., 2023). Although many people refer to ‘spirituality’ in the context of the Camino, it is such an ambivalent term that its meaning is unclear, and it is often interpreted in a reductionist way. For some, spirituality is simply *slow tourism* (Fullagar et al., 2012), but distinguished by the ‘destination’, and not by the journey or the walking itself. This raises the key question: What makes a given journey spiritual? Is it because of rituals, as suggested by Turner et al. (1969), or simply visiting some cultural or religious artefact?

It seems therefore that ‘spirituality’, used in many texts on the Camino, requires further reflection in order to understand a series of references that appear during the pilgrimage to Santiago (Brumec, 2023; Puchalski et al., 2014; Roszak, 2023a, 2023b). Does the term ‘spiritual’ mean ‘allied’ and an extension of the religious, or is it rather the counterfeit that appears, replacing religion, in the spirit of the ideology of the ‘woke’? In what sense is the road to Compostela a spiritual road?

To answer this question, we first try to argue that there are two narratives, one that perceives the religion/spirituality antagonism (Kallo, 2024) – or is it rather an extension of religion, with its specific language and mode of expression? As a start it is worth recalling what is understood by the term ‘spiritual’ in classical theology and apply it to the Camino, and finally to systematise the most important dimensions of the spirituality of the pilgrimage to Santiago.

## Meaning of the ‘Spiritual’ in Christian Theology

It sometimes happens that a certain term is used for a long time and acquires different characteristics in different epochs, and as a consequence its original meaning is often forgotten. A good example is the term *mysticus*, which today is associated with states of ecstasy but for mediaeval theologians was synonymous with *mysterium*, i.e. the mystery before which contemplative silence is maintained (Poirel, 2021). The situation is similar with the term ‘spiritual’, which, like pilgrimage, is undergoing an important semantic inflation, probably under the influence of the philosophy of a given epoch. Nevertheless, in the theological literature one can find the phrases ‘spiritual well-being’ or references to the spiritual life, which is made up of the virtues, which perfect the intellect and the will. Furthermore, one often speaks of spiritual theology, which is the practical implementation of the postulate of ‘living according to the spirit’ (cf. Rom 8,5). So what does the term ‘spiritual’ mean from a philosophical and theological perspective? It is worth referring to Thomas Aquinas, who presents a classical position on the understanding of this term.

### Philosophical and Theological Foundations of Spirituality

In Thomas Aquinas’ thought, the adjective ‘spiritual’ means – strictly speaking – to be separated from matter; therefore, substances separated from matter are called spiritual. In a broader sense, the term also means to be detached from matter, when, for example, certain material substances exceed to a greater or lesser extent their dispositional qualities of matter. In this sense, spirituality is a characteristic of the activities by which living beings, including people, carry out soul operations until the overcoming occurs and even a corporeal organ is no longer needed (Margelidon & Floucat, 2011). In this way, spirituality is related to the degree of immateriality, and its growth is related to freeing oneself from material limitations, thus achieving the universal. Colour is ‘spiritual’ in relation to the material entity that sustains it. Aquinas (1954, Quaestiones disputatae de veritate, q. 3 a. 1 ad 2) even speaks of ‘*esse spirituale vel immateriale*’, referring to the mental representations of the objects it learns.

Broadening the perspective of understanding the spiritual beyond metaphysics and epistemology, it is worth noting that in Thomas Aquinas’ terminology, *spiritualitas* means the opposite of the temporal and is synonymous with spiritual life, which brings the spiritual fruits (*fructus spiritualis*). This relates to the sanctification brought about by the sacraments, which is achieved by grace, through participation in the *perfect spiritualitas* of Christ. In the moral life, spirituality is associated with moderation *(continentia*), abstinence, dedication or some dimension of the life of God himself (Margelidon & Floucat, 2011).

At the same time, for Aquinas this spiritual life is an intellectual life, which is not based on any other, but is a life in itself, per se. The body is alive, though it is not life itself, but a participant in it; therefore, the spiritual life is the ‘first life’ which is drawn from the very source of life itself (Aquinas, 2010, Super Io., cap. 5 l. 4).

### Materialise the Spiritual and Spiritualise the Material

There is much discussion around this understanding of the spiritual, which attempts to avoid the Manichean inclination that pits the spiritual and the material against each other. It is worth noting that the descriptions of miracles in the *Codex Calixtinus* show God at work in matter, in the midst of everyday events. The Way shows precisely the power of God at work in such a world. Interestingly, these miracles show the Apostle St James struggling with a lack of self-esteem, giving encouragement, but also helping to overcome the pilgrim’s depression. Thus, the miracles are about spiritual matters, not simple prescriptions to improve physical health. The medicine offered by the miracle goes much further, because it is the healing of the human spirit that then affects the body and the way we live in the world.

This shows that spirituality is best perceived through the prism of the Thomistic view of man as a corporeal/spiritual unity, in the categories of the theory of hylemorphism. It is about perceiving body and soul as matter and form (following Aristotle’s terminology), potentiality and action, respectively, interconnected and not antagonised.

It often seems that ‘Manichean’ thinking takes over the approach to pilgrimage, when only what is opposed to the body is treated as spiritual: not only ‘separate’, but even ‘hostile’. Man then becomes a stage for the struggle of attrition instead of for the effort of harmonisation. The consequences of this approach to spirituality lead to the separation of the dimensions of pilgrimage, which are organically connected to each other. It is significant that for Thomas Aquinas, in the case of man, the spiritual does not exist ‘apart from’, but ‘through’ the material. Thus, for St Thomas, even walking or other physical activities can be a spiritual experience (Roszak & Gutowski, 2021).

This hylemorphism of pilgrimage, i.e. seeing the material of a journey as a way for man to reach the spiritual because of his ontological constitution, has practical consequences for shaping the spirituality of the pilgrim. The dialectical approach to the matter-spirit relationship, with all its diversity, gives way to the scholastic practice *per materiam and Spiritum*, from matter to spirit. This is achieved with reference to the goal, when the material activities performed become a means to approach the final goal (Gudaniec, 2024). As Jesús Tanco observes, ‘religion, properly understood, has to materialise the spiritual in concrete acts that affect what, in justice or for love’s sake, must be given to God. But on the other hand, we must spiritualise the material, knowing that everything we do and the way we do it in this earthly life has merit and influences eternal happiness’ (Tanco, 2024).

But this has consequences that affect matter: the spiritual development of man who imitates God brings him spiritual vision without ceasing to be material. It is a matter of gaining a certain *connaturalitas*, an intimacy with the spiritual, in order to develop the ability to see – even in the midst of material events – what they bring with them spiritually (Huzarek, 2021).

### Religion VS The Spiritual

In the postmodern paradigm, we can see another phenomenon of trying to redefine what is religious by reducing it to one of its manifestations. In the spirit of evolutionary biology, religion is reduced to a ‘by-product’ of evolution, to satisfying needs, to maintaining views. Religion is seen as a ‘means’ to a selfish end, opposed to spirituality. This dichotomy is based on an institutional and personal distinction, but at its core, it resembles the gnosis of the second century, which reduced the valuable to the spiritual, segregating people into corporeal and *pneumatikoi*. Christianity opposed such a dialectical approach, emphasising the value of the body and rejecting the egalitarian interpretation of Revelation as being reserved only for the prominent disciples of Jesus.

However, the distinction between religion and spirituality was only formed in modern times, because during the heyday of the pilgrimages to Compostela, there was no general concept of ‘religion’, but a virtue-based approach to it (*vera religio* is true religiosity, not a question of which religion is true). Contrary to our contemporary intuitions, there was no tension where today we see supposed conflicts or rivalries. Religiosity was a virtue, an ability that could grow, and it consisted in assigning everything to God (*ordo ad Deum*) (Roszak & Gutkowski, 2021). Religion is therefore a relational virtue that connects all earthly affairs to the ultimate end, showing its deep meaning in the context of eternity. He did not deprive the meaning of the temporal, but looked for ultimate references.

However, to understand the difference and the connections it is necessary to understand not only what religion was, but also spiritual life. The spiritual was related to the virtues, faith, charity and the sacraments. It considered how the spiritual life is ‘lost’ and when it grows through the virtues (Aquinas, 2020, Quodl. VII, q. 7 a. 1 ad 5), especially charity, which is what the spiritual life consists of (Aquinas, 1983, De malo, q. 14 a. 2 co). Therefore, the intensification of love for God and others is a manifestation of the development of the spiritual life; and since love concerns the good and is realised according to a certain order (*ordo caritatis*), the ordering of the world of loves is related to spirituality. The means to reach the perfection of the spiritual life is to deny the inferior by putting the love of God in the first place. Therefore, spiritual progress is possible by abandoning not only external goods, but also that which the person loves most (i.e., his own will). The spiritual path is associated with not relying on one’s will or the course of the pilgrimage, but relying on providence, which is God’s way of leading to the goal. This is related to the *disciplina spiritualis*, which is a way of learning about what is valuable and good, something hidden like the treasure in the field in Jesus’ parable and which requires a proper assessment between temporal and eternal prosperity (as in the case of Job’s tale).

For Thomas Aquinas, the spiritual life is related to faith, but this does not mean exclusivity, because this spiritual life is also manifested in the possession of other virtues, although charity remains its cornerstone. Ultimately, these activities find their model in Christ; this is why Thomas Aquinas sees the significance of this spiritual life in ‘planting’ the life of Christ in the believer, which is the task of faith (Aquinas, 2012, *Super II Cor*., cap. 4 l. 4), since Christ dwells in the believer through faith (cf. Eph. 3:17). Therefore, the spiritual life is a manifestation of a certain dynamism, but a unique kind, because it strives for ‘spiritual peace’ (*quies spiritualis*). Living a spiritual life means acquiring and remaining in that specific peace, which is not passivity, but an effort to replant life.

From this perspective, one can understand the number of approaches to the relationship between religion and spirituality that have been presented in recent years. According to B. Welte, it was pointed out that sometimes there can be a lack of the essence of religion, i.e. external forms or rituals devoid of content (Doburzyński, 2021). This results from followers performing gestures unconsciously and externally, but without accepting the content. They may be fulfilled by tradition or custom, but the religious content is postponed or changed. The pilgrimage is then practised in its form, but without the background that has given it this form over the centuries.

In pilgrimage research, a postmodern perception of pilgrimage, linked to the experience of enlightenment, transformation and life change, is gaining ground. This search for a meaningful experience is therefore increasingly present in tourism, where the spiritual quest is mentioned, i.e. the search for one’s identity and contact with oneself, others and the world (Collins-Kreiner, 2016). Pointing to the spiritual ‘instead of’ the religious, according to Collins-Kreiner, means emphasising the individual, subjective experience of the sacred and abandoning the institutionalised form of religious experience, in which authorities play an important role. Spirituality perceived in this way is associated with peace resulting from the development of beliefs about the meaning of life. Therefore, as Vincett and Woodhead (2016) emphasise, spirituality thus perceived began to be treated as an alternative to religion because it wants to discover the divine beyond the traditional paradigm (belief and practice), in favour of a functional approach with three threads: meaning in life, being connected and transcendence.

However, Michèle M. Schlehofer, Allen M. Omoto and Janice R. Adelman (Schlehofer et al., 2008) point out that although religiosity is associated with community affiliation, personal beliefs and organisational practices, spirituality is more abstract in its concepts than religion, and therefore includes non-theistic concepts of the absolute. The spiritual experience of a pilgrimage is also often spoken of in terms of one level; thus, the physical aspects and the spiritual and religious aspects constitute overlapping layers.

Reflections on the relationship between spirituality and religion today raise many questions: whether spirituality is a consequence of religiosity or a path leading to it. As Domazet (2022) points out,

the word ‘spirituality’ has become polysemous and obsolete. That is, the ‘return of spirituality’ revealed the progressive weakening of the meaning of the Church and its spirituality, as well as modern man’s thirst for inner well-being and spiritual values. However, this enthusiasm for spirituality coincides with the loss of the prophetic, social and political dimensions of faith and its complete privatisation. This is why the German theologian J.B. Metz draws a clear dividing line between spirituality in postmodern times and Christian spirituality, which he calls ‘mysticism with open eyes’.

However, we are facing a ‘second wave’ of research on the connection between lived spirituality and religion, which seeks a new framework for further research. The first wave focussed on the reasons for separating society from religion and moving towards spirituality, whilst the second wave tried to situate spiritual practices within a specific social context, without losing the relationships. Attempts are now being made to see where the differences and mutual dependencies lie.

## What is Spiritual About the Camino According to Pilgrims?

When analysing written accounts of pilgrimages, in the form of diaries and books, it can be observed that the term ‘spiritual’ refers to one’s experiences during the pilgrimage. One of the recent surveys, conducted by Michael Basil (2023), highlights that for many pilgrims the differences between tourism and pilgrimage are disappearing. Among the effects of pilgrimage they indicated a break from everyday activities and a simpler life, a slower pace of life, a sense of connection with nature and neighbours, a sense of fulfilment in life and a strengthening of their personal identity. However, this says little about their motivations, as it is more about the results. It seems that the research does not take this distinction between motives and fruits sufficiently into account.

Research by Suzanne Amaro, Angela Antunes and Carla Henriques (Amaro et al., 2018) (on a group of 1,140 respondents from 45 countries) followed this direction and concluded that the most common motives were spiritual (followed by new experiences, nature and sport, and finally religious ones). In this case, religiosity was perceived more psychologically, as a more or less conscious decision-making factor. If someone goes to church for Mass and justifies it by the search for peace and well-being, does this contradict the religious motive that is the source of this peace and well-being (Han et al., 2024)? It turns out that the very choice between the options can be wrong, as if one were to ask whether someone goes to a football match for the sporting value or for the thrill of cheering their team on. Research proposals by Snežana Brumec, Miran Lavrič and Andrej Naterer (Brumec et al., 2023) point in this direction, highlighting the absence of ‘purely religious’ motivations, but finding mixed ones. These changes in pilgrims’ statements, as well as the differences between young and old, were already noticed in the 1990s when pilgrimages to Lourdes (Van Uden, 1991) as well as Santiago (Mróz, 2021; Nelson-Becker et al., 2023) were analysed.

### Discovering the Meaning

Among the accounts of the pilgrimage to Santiago, a deep reflection on the meaning of life and the effort made, which is achieved by entering into silence, into the rhythm of the path and away from ‘objectification’, are often noted as spiritual moments. Seeing people/entities in the world of the products of civilisation and establishing relationships is remembered as a moment of spiritual experience. This is how Z. Pucia puts it in *The Way of Faith* [Droga wiary], stressing the role of humility, which means not putting oneself in the centre and focussing on one’s own experiences (Pucia, 2019). This detachment from oneself and seeing others is a spiritual experience that requires not being deafened by the noise of the world. This relates to the category of ‘spiritual knowledge’, which penetrates the external, uncovering the meaning of many invisible phenomena in everyday life.

This discovery of meaning can also have a historical dimension: encounters with places sanctified by the presence of previous generations of pilgrims awaken or revive that narrative and ideal (Wooding, 2013).

### Pilgrims’ Spiritual Experience

Another publication reporting on the pilgrim experience, Shirley MacLaine’s (2001) *The Way a Spiritual Journey*, points out the dichotomy of the physical and the spiritual, focussing on the choices made by the pilgrim. It is therefore not about isolation and escape, or breaking away from dependence on things (electronic ‘cordons’, disconnection, are constantly emphasised), but a different perspective on what one lives. It is a process of spiritual ordering that ultimately brings peace when one reaches the destination. This means that there are, so to speak, parallel dimensions – movement in space and spiritual progress – themes that have always been characterised by tension in pilgrimage theology (think of *modern devotio* and contemporary cyber-pilgrimages).

The sign of undertaking this spiritual journey is the pilgrim’s blessing (Roszak & Seryczyńska, 2020) which is not a simple desire, but a sending forth: ‘the blessing of sending forth is to place oneself in God’s hands, to follow a marked path, but in its inner experience, rather than in an external itinerary, a way of moving and travelling quite different from curiosity, tourism or wandering without a goal. Not just any road or walk is the “Way of St. James” or the “walk to the tomb of the Apostle”’ (Buide del Real, 2022).

Pilgrims’ memoirs and diaries mention spiritual renewal, spiritual vision of material reality and the emergence of order. They describe how physical activities such as walking/knowing/feeling/experiencing become spiritual. The concept of the pilgrim’s ‘spiritual experience’ becomes indefinite. What does it really mean? It can point towards a deep knowledge, as opposed to superficial knowledge, involving the soul, developing its capacities to know and to want what the ‘virtues’ consist of (Serra Pérez, 2023). Furthermore, it seems that for many pilgrims spirituality is associated with hearing a ‘call’, i.e. a deep motivation to walk the path. This focus on the ‘intention’ for which one sets out – this determines the nature of this journey, for it is an ‘end’ that shapes the ‘means’. The rituals that the pilgrim undertakes support this fundamental orientation.

## Spiritual Dimensions of the Way of St James

After presenting the theological understanding of the spiritual, the relationship with religiosity and the reference to the Way as such, it is worth dwelling on the term ‘spiritual way’ used to refer to this route. What does its spiritual being consist of? From a theological point of view, several dimensions of the spiritual understanding of the pilgrimage can be identified.

### Transformative (penitential) journey

The way is a spiritual way in the sense that it restores *salus*, i.e. spiritual health and thus salvation, and this key term for Christianity is synonymous with freedom, both as freedom ‘from’, i.e. to be saved from danger, but also as freedom ‘to’, i.e. the ability to achieve one’s goal (Oviedo, 2024).

In this sense, pilgrimage is a penitential experience, not only as expiation for an evil committed, but as a key to the virtue of penance, which Thomas Aquinas defined as the capacity to grieve for wrongdoing, with the intention of isolating oneself from it (Aquinas, 2014, Summa Theologiae III, q.85, a.1c). As an act of will, it is not only the cessation of evil, but also a reward, a return to justice, to relationships, and therefore a sacrifice, for the essence of sacrifice is the recognition of God as the source from which everything proceeds, and this is emphasised by the things offered to him. Therefore, the fruit of penance is to regain full freedom, and this is realised through relationships over which there is no longer a shadow of evil (Vijgen, 2015). ‘Penance opens the door to the other virtues by expelling sin through the virtue of faith and charity, which are naturally prior. And in such a way it opens the door for them that they themselves enter at the same time with it’ (Aquinas, 2014, Summa Theologiae III, q.85, a.6, ad 3).

In the classical approach to pilgrimage in their *Image and Pilgrimage in Christian Culture*, Edith and Victor Turner treat it as an expression of the journey from the secular to the spiritual world and vice versa. In their model, they emphasise that the key to understanding pilgrimage is the transformation of oneself in confrontation with the transcendent powers, i.e. God, Mary or the saints. This translates into moving away from the local, well-known and dominant world, towards the experience of liminality that leaves a deep imprint on the personal life of the pilgrim. Thus, among the changes that are called spiritual, there are those that are therapeutic, that is, liberating from depression, loss or trauma. Such transformation includes a mental change, a prosocial attitude that characterises spirituality and religion, and at the same time constitutes the so-called ‘path effect’ (Brumec, 2021).

Significantly, many studies on pilgrimages emphasise the psychological effect of pilgrimage as a cure for anxiety and depression, controlling negative emotionality, which is also associated with a reduction of frontal symmetry when processing religious stimuli in pilgrims (Kéri, 2023). This effect was not observed in those who walked the same route for non-religious reasons.

### Reflective journey

Charles Taylor, in describing the emergence of the secular age, highlighted the processes associated with the breakdown of the dualism that had characterised earlier times. When this dualism no longer adequately describes the present moment, there remains a multitude of spiritual possibilities from the destruction of what came before. It makes one question one’s own identity in the face of so many others. Taylor calls this the *nova effect* and describes it as follows:

When … the other becomes more and more like me, in everything but faith: same activities, professions, opinions, tastes, etc., then the question posed by the difference becomes more insistent: why my way and not hers? There remains no other difference that makes change absurd or unimaginable. (Taylor, 2007)

The pilgrimage becomes spiritual when the relationship with the destination of the pilgrimage – the visit to the tomb of St. James the Apostle in Santiago de Compostela becomes a relationship with what the Apostle represents and, therefore, with integration with the Church.

The spiritual guide will therefore mark out the path that will lead to this effect: spiritual empowerment. Recovering relationships and inner order means strengthening inner faith, acceptance, resilience and spiritual discipline, thanks to which it is possible to cope with adversity (Hilario and Sy Su, 2023). And this leads to spiritual fulfilment, as reported by pilgrims returning from the Camino (Baek et al., 2022).

### Transcendental Journey

Pilgrimage is also a spiritual journey because it transcends the immediacy of experience. It is about moving from different levels of experience to noticing growing needs, not only material (food and accommodation), but spiritual (Horvat & Pavlic, 2020). Archbishop Julian Barrio, Archbishop Emeritus of Santiago, pointed this out in his letters, seeing the journey as a journey of transcendence, transcending the dimensions of description and asking questions that have no other place (Mizdrak, 2024). This spiritual face can then be described as affective, but not in the sense of feelings, because it is more than passions and there is a difference between love and charity. *Affectus* describes the movement of the will in discovering the near good, thus integrating intellect and will.

In this context, it is worth remembering and relating to the path the three types of existence that Soren Kierkegaard distinguished: aesthetic, ethical and religious. This Danish philosopher fought against an idealistic/rationalistic view of truth that lacked existential roots, which would lead to despair, to being one of many, a *nobody* instead of *someone*. In an *egocracy* or egolatry, a pilgrimage can play a restorative role for relationships; in this sense, spirituality will be a liberation of the spirit from the limitations of the endogamous culture. It is a transition from the ‘aesthetic’ level of life, when one is immersed in what pleases, to the ‘ethical’ level, when one is concerned with what is of value, to finally reach the ‘religious life’, which is to trust and rely on what is the ultimate goal. In this way, to transcend in pilgrimage means not only to overcome one’s own limits, to see ‘beyond’, but to reach the final goal.

Snezana Brumec (2024) analysed in detail the experience of transcendence during the pilgrimage, recalling the concept of ‘self-spirituality’, which refers to a person’s inner world, their true self, leading to self-realisation, which can be achieved by regaining the relationship with the true self; this is achieved through ‘feelings of self-transcendent connectedness’ (something that resembles the sense of *communitas*). Referring to Watts’ Marcus, Brumec sees the path as an inner spiritual exploration. Spiritual growth takes place in five stages in which four fundamental concepts emerge: the realisation that true freedom and contentment are separate from material possessions, the role of meditation and spirituality, the fostering of deep connections, and the use of meaningful suffering as a conduit for awakening. This leads to the so-called Fluid Spirituality, thus transcending materialism and consumerism.

## Study Limitations

The reflections on the phenomenon of Camino de Santiago pilgrimage as a ‘spiritual journey’ and the impact of these walks on human spiritual well-being presented in the following article are based on the Thomistic understanding, in the case of man, the spiritual does not exist ‘apart from’, but ‘through’ the material. For Thomas Aquinas, physical activities – including walking – can be a spiritual experience. ‘Spirituality’ – a spiritual motif used in dozens of tesents, reports, blogs related to the Camino de Santiago requires further reflection on the understanding of the ‘spiritual journey’ to the tomb of St James. Indeed, the term ‘spiritual’ is used ambivalently in many of these, and is often interpreted in a reductionist manner.

## Conclusions

Treating the Way of St James as a spiritual journey is done in two basic ways: either as an introduction to religious experience (from spirituality to religion) or as the development of a previous religious motivation (from religion to spirituality). Both cases, although very different, are described in the category of ‘spiritual and religious’ motivations about which pilgrims are asked in Santiago. Although these are levels of experience that may be related, we hear more and more pilgrims talking about spirituality without the Church, atheistic spirituality, and as we have noted, this means a reductionist understanding of spirituality. Will such a spiritual transformation take place, during any walk? It seems that this is not the case, that not only the material circumstances are decisive, not only the ‘matter’, but also the ‘form’. The theological answer points to grace, which, like Christ on the road to Emmaus, joins those who start the journey and seek answers that are not partial and immanent, but holistic and transcendent.

## References

[CR1] Amaro, S., Antunes, A., & Henriques, C. (2018). A closer look at Santiago de Compostela’s pilgrims through the lens of motivations. *Tourism Management,**64*, 271–280. 10.1016/j.tourman.2017.09.007

[CR2] Aquinas, T. (1954). *On Truth*. 3 vols., trans. Mulligan, R. W., Mc Glynn, J. V., and Schmidt, R. W. Henry Regnery Company.

[CR3] Aquinas, T. (2010). *Commentary on the Gospel of John, Books 1–5*. Transl. Larcher F. R., and. Weisheipl J. A. Catholic University of America Press.

[CR4] Aquinas, T. (2012). *Commentary on the letters of Saint Paul to the Corinthians*. The Aquinas Institute for the Study of Sacred Doctrine.

[CR5] Aquinas, T. (2014). *The Summa Theologica*. Catholic Way Publishing.

[CR6] Aquinas, T. (1983). *Disputed Questions on Evil*. Oesterle, J.T. Notre Dame University Press.

[CR7] Aquinas, T. (2020). *Thomas Aquinas’s Quodlibetal questions*. Oxford University Press.

[CR8] Baek, K., Choe, Y., Lee, S., Lee, G., & Pae, T.-I. (2022). The Effects of Pilgrimage on the Meaning in Life and Life Satisfaction as Moderated by the Tourist’s Faith Maturity. *Sustainability,**14*, 2891. 10.3390/su14052891

[CR9] Basil, M. (2023). Motivations and meanings for Camino pilgrims. *Tourism Recreation Research,**48*, 1–12. 10.1080/02508281.2023.2274156

[CR10] Brumec, S. (2021). The Camino de Santiago in Late Modernity: Examining Transformative Aftereffects of the Pilgrimage Experience. *International Journal of Religious Tourism and Pilgrimage,**9*(6), 1–13.

[CR11] Brumec, S. (2022). Life changes after the Camino de Santiago pilgrimage, including a deeper sense of spirituality. *Journal for the Study of Spirituality,**12*(1), 20–35. 10.1080/20440243.2022.2042948

[CR12] Brumec, S. (2023). Spirituality of Pilgrims on the Camino de Santiago: Existential Questions and the Meaning of Life. *Qeios*. 10.32388/7M8AEV

[CR13] Brumec, S. (2024). Spirituality in Late Modernity: Exploring the Tenets of Spirituality of Camino de Santiago Pilgrims. *The International Journal of Religion and Spirituality in Society,**14*(4), 89–120.

[CR14] Brumec, S., Lavrič, M., & Naterer, A. (2023). Examining Motivations to Walk the Camino de Santiago: A Typology of Pilgrims. *Pastoral Psychology,**72*, 479–500. 10.1007/s11089-023-01071-1

[CR15] Buide del Real, F. (2022). Espiritualidad del Camino. La actualidad espiritual del Calixtino hoy [Spirituality of the Camino. The spiritual actuality of the Calixtinus today]. *Revista De La Catedral De Santiago,**6*, 27–31.

[CR16] Cazaux, F. (2011). To be a pilgrim: A contested identity on Saint James Way. *Tourism. an International Interdisciplinary Journal,**3*(1), 353–367.

[CR17] Collins-Kreiner, N. (2016). The lifecycle of concepts: The case of ‘Pilgrimage Tourism.’ *Tourism Geographies,**18*(3), 322–334. 10.1080/14616688.2016.1155077

[CR18] Doburzyński, D. (2021). *Znak drogi. Teologia pielgrzymowania z perspektywy Camino de Santiago* [*Sign of the Way. A theology of pilgrimage from the perspective of the Camino de Santiago*]. Wydawnictwo Naukowe Uniwersytetu Mikołaja Kopernika.

[CR19] Domazet, J. (2022). Ecclesiological Themes in the Pontificate of Pope Francis. *Crkva u Svijetu,**57*(4), 605–636.

[CR20] Faria, C. A. P., Remoaldo, P. C., & Martins, M. F. S. V. (2024). Landscape and Senses in a Portuguese Municipality on the Way of St. James: Potential Impacts on the Well-Being of Pilgrims. *Journal of Religion and Health,**63*, 133–158. 10.1007/s10943-022-01617-2 35925457 10.1007/s10943-022-01617-2 PMC9362657

[CR21] Feliu-Soler, A., Pérez-Aranda, A., Luciano, J. V., Demarzo, M., Mariño, M., Soler, J., Van Gordon, W., García-Campayo, J., & Montero-Marín, J. (2021). Psychometric properties of the 15-item five facet mindfulness questionnaire in a large sample of spanish pilgrims. *Mindfulness,**12*(4), 852–862. 10.1007/s12671-020-01549-6

[CR22] Felkai, P. (2019). Medical Problems of Way of St. James Pilgrimage. *Journal of Religion and Health,**58*, 566–571. 10.1007/s10943-018-00744-z 30604326 10.1007/s10943-018-00744-z

[CR23] Fullagar, S., Markwell, K., Wilson E. (Ed.) (2012). *Slow Tourism: Experiences and Mobilities*. Channel View Publications.

[CR24] Gudaniec, A. (2024). Personal Experience of Suffering: Reflections Inspired by Elements of Karol Wojtyła’s Philosophical Anthropology. *Scientia Et Fides,**12*(1), 215–229.

[CR25] Han, J. W., Kim, M. J., Chung, N., & Kim, J. S. (2024). The effect of the authenticity of the Catholic pilgrimage site on spiritual well-being and quality of life of pilgrims. *International Journal of Tourism Research,**26*(1), e2634. 10.1002/jtr.2634

[CR26] Harris, M. B. (2019). The Physiological Effects of Walking Pilgrimage. *International Journal of Religious Tourism and Pilgrimage,**7*(1), 85–94.

[CR27] Harris, M. B., & Wolf, M. R. (2013). Cardiovascular disease risk following a 758 km pilgrimage. *International Journal of Sports Medicine,**34*(8), 727–731. 23382008 10.1055/s-0032-1331172

[CR28] Hernández, A. (2022, June, 10). Responsabilidad Social Corporativa 400 empleados de Cisco llegan a Santiago junto al ‘robot peregrino’. Press Release [Corporate Social Responsibility 400 Cisco Employees Arrive in Santiago with the 'Pilgrim Robot'. Press Release]. https://news-blogs.cisco.com/emea/es/2022/06/10/400-empleados-de-cisco-llegan-a-santiago-junto-al-robot-peregrino/

[CR29] Hilario, R. C., Sy, Su., & C.C. (2023). The Efficacy and Limits of Pilgrimage as Therapy for Depression. *Religions,**14*(2), 1–13. 10.3390/rel14020181

[CR30] Horvat, S., & Pavlic, R. (2020). Teološka antropologija pred izazovom (evolutivne) kognitivne znanosti o religiji [Theological anthropology before the (evolutionary) cognitive science of religion]. *Diacovensia,**28*(3), 303–317.

[CR31] Huzarek, T. (2021). The theological sources of human identity. *European Journal of Science and Theology,**17*(6), 29–38.

[CR32] Kallo, A. (2024, January 17). Around 4 in 10 Americans have become more spiritual over time; fewer have become more religious. https://www.pewresearch.org/short-reads/2024/01/17/around-4-in-10-americans-have-become-more-spiritual-over-time-fewer-have-become-more-religious/?utm_content=buffer15706&utm_medium=social&utm_source=twitter.com&utm_campaign=buffer-pew

[CR33] Kéri, S. (2023). *Frontal Asymmetry in Pilgrims. Religions,**14*(8), 1072. 10.3390/rel14081072

[CR34] Klimiuk, J., & Moriarty, K. J. (2021). The Lourdes Pilgrimage and the Impact on Pilgrim Quality of Life. *Journal of Religion and Health,**60*, 3775–3787. 10.1007/s10943-021-01398-0 34505260 10.1007/s10943-021-01398-0 PMC8428497

[CR35] Latusi, S., & Fissore, M. (2021). Pilgrimage routes to happiness. Comparing the Camino de Santiago and Via Francigena. In A. Riani Costa Perinotto, V. Feder Mayer, J. R. Rodrigues Soares (Eds.), *Rebuilding and restructuring the tourism industry: Infusion of happiness and quality of life* (pp. 157–182).

[CR36] MacLaine, S. (2001). *The Camino: A Journey of the Spirit*. Atria Books.

[CR37] Margelidon, Ph-M., Floucat, Y. (2011). *Dictionaire de philosophie et de theologie thomistes*. Parole et Silence.

[CR38] Margry, P. J. (2015). To Be or not to Be… a Pilgrim. Spiritual Pluralism Along the Camino Finisterre and the Urge for the End. In C. Sánchez-Carretero (Ed.), *Heritage, Pilgrimage and the Camino to Finisterre. Walking to the End to the World*, (pp. 175–211). Springer.

[CR39] Mizdrak, I. (2024). Philosophical Significance of the Way, Experience, and Silence in the Context of Camino de Santiago. *Verbum Vitae,**42*(1), 17–37.

[CR40] Morris, P. A. (1982). The effect of pilgrimage on anxiety, depression and religious attitude. *Psychological Medicine,**12*(2), 291–294. 10.1017/S0033291700046626 7100352 10.1017/s0033291700046626

[CR41] Mróz, F. (2020). Poles Travelling to Compostela in Time and Space. *Journal of Cultural Geography,**38*(2), 206–234. 10.1080/08873631.2020.1864086

[CR42] Mróz, F. (2021). The Impact of COVID-19 on Pilgrimages and Religious Tourism in Europe During the First Six Months of the Pandemic. *Journal of Religion and Health,**60*, 625–645. 10.1007/s10943-021-01201-0 33611686 10.1007/s10943-021-01201-0 PMC7896876

[CR43] Mróz, F., Mróz, Ł, & Krogmann, A. (2019). Factors conditioning the creation and development of a network of Camino de Santiago routes in Visegrad Group countries. *International Journal of Religious Tourism and Pilgrimage,**7*(5), 56–71.

[CR44] Nelson-Becker, H., Pickard, J., & Sichling, F. (2023). Adventure and Spiritual Restoration: Older Adult Motivations for Undertaking a Pilgrimage on El Camino de Santiago. *Journal of Gerontological Social Work,**66*(6), 822–838. 10.1080/01634372.2023.2181906 36809992 10.1080/01634372.2023.2181906

[CR45] Ollivier, B. (2011). *Życie zaczyna się po sześćdziesiątce* [*Life begins at sixty*]. Wydawnictwo Literackie.

[CR46] Oviedo, L. (2024). Evolutionary Explanations of Pain and Suffering: A ‘Gift to Theology’ or a Challenge. *Scientia Et Fides,**12*(1), 89–105.

[CR47] Platovnjak, I., & Zovko, V. (2023). Human Bodily Movement and Spirituality. *Nova Prisutnost: Časopis Za Intelektualna i Duhovna Pitanja,**21*(3), 541–556.

[CR48] Poirel, D. (2021). *Existe-t-il une mystique au Moyen Âge* [*Is there such a thing as mysticism in the Middle Ages*]. Brepols.

[CR49] Puchalski, C. M., Vitillo, R., Hull, S. K., & Reller, N. (2014). Improving the spiritual dimension of whole person care: Reaching national and international consensus. *Journal of Palliative Medicine,**17*(6), 642–656. 10.1089/jpm.2014.9427 24842136 10.1089/jpm.2014.9427 PMC4038982

[CR50] Pucia, Z. (2019). *Droga wiary. Camino Frances* [*The journey of faith. Camino Frances*]. Głogowiec.

[CR51] Roszak, P. (2019). Sacred and Space in Post-Secular Pilgrimage: The Camino de Santiago and Relational Model of the Sacred. *International Journal of Religious Tourism and Pilgrimage,**7*, 33–40.

[CR52] Roszak, P. (2023a). From Tourist to Pilgrim: Theological and Pastoral Challenges in the Context of the Camino de Santiago. *Pastoral Psychology,**72*, 647–657.

[CR53] Roszak, P. (2023b). Not Only Coping: Resilience and Its Sources from a Thomistic Perspective. *Journal of Religion and Health,**62*, 2734–2745. 36454332 10.1007/s10943-022-01711-5 PMC10366280

[CR54] Roszak, P., & Gutowski, T. W. (2021). Pobożność jako cnota relacyjna wg św. Tomasza z Akwinu [Piety as a relational virtue according to St. Thomas Aquinas]. *Studia Gdańskie,**48*, 93–103.

[CR55] Roszak, P., & Seryczyńska, B. (2020). A pilgrim blessing – An alluring folklore or expression of piety? Theological insights from the Camino de Santiago. *Bogoslovni Vestnik,**3*(80), 685–696.

[CR56] Schlehofer, M., Omoto, A., & Adelman, J. (2008). How Do “Religion” and “Spirituality” Differ? Lay Definitions among Older Adults. *Journal for the Scientific Study of Religion,**47*(3), 411–425. 10.1111/j.1468-5906.2008.00418.x

[CR57] Serra Pérez, M. A. (2023). Fundamento metafísico y ético de la educación: las passiones animae de Tomás de Aquino y Veritatis Splendor [Metaphysical and ethical foundation of education: the passiones animae of Thomas Aquinas and Veritatis Splendor]. *Cauriensia. Revista Anual De Ciencias Eclesiásticas,**18*, 399–418.

[CR58] Seryczyńska, B. (2019). Creative Escapism and the Camino de Santiago. *International Journal of Religious Tourism and Pilgrimage,**7*(5), 48–55.

[CR59] Silva, F. M., Braga, J. L., Otón, M. P., & Borges, I. (2023). Pilgrimages on the Portuguese Way to Santiago de Compostela: Evolution and Motivations. *Religions,**14*, 1017. 10.3390/rel14081017

[CR60] Tanco, J. (2024). Aspectos religios del Camino de Santiago [Religious aspects of the Camino de Santiago]. https://jesustancolerga.wordpress.com/

[CR61] Taylor, Ch. (2007). *A Secular Age*. Harvard University Press.

[CR62] Turner, V., Abrahams, R., Harris, A. (1969). *The Ritual Process: Structure and Anti-Structure* (1st ed.). Routledge. 10.4324/9781315134666

[CR63] Tykarski, S., & Mróz, F. (2024). The Pilgrimage on the Camino de Santiago and Its Impacts on Marital and Familial Relationships: An Exploratory Study. *Journal of Religion and Health,**63*, 109–132. 10.1007/s10943-023-01825-4 37126119 10.1007/s10943-023-01825-4 PMC10150338

[CR64] Van Uden, M. (1991). Modern Pilgrimage and Faith. *Journal of Empirical Theology,**4*, 32–51.

[CR65] Vijgen, J. St. (2015) Thomas Aquinas and the Virtuousness of Penance. On the importance of Aristotle for Catholic Theology *Nova et Vetera. English edition, 13(2)* 601–616

[CR66] Vincett, G., & Woodhead, L. (2016). Spirituality. In L. Woodhead, Ch. Partridge, & H. Kawanami (Eds.), *Religions in the Modern World Traditions and Transformations* (pp. 323–344). Routledge.

[CR67] Vistad, O. I., Øian, H., Williams, D. R., & Stokowski, P. (2020). Long-distance hikers and their inner journeys: On motives and pilgrimage to Nidaros, Norway. *Journal of Outdoor Recreation and Tourism,**31*, 1–10. 10.1016/j.jort.2020.100326

[CR68] Wooding, J. (2013). Historical-Theological Models of Pilgrimage as a Resource for Pilgrimage Tourism. *Journal of Tourism Consumption and Practice,**5*(2), 61–72.

[CR69] Zhang, K., Ramos-Riera, I., & Labajo, V. (2024). Feet in Hell, Spirit in Heaven: Spiritual Transformation of Chinese Travelers on the Camino de Santiago. *Journal of Religion and Health,**63*, 89–108. 10.1007/s10943-023-01976-4 38147260 10.1007/s10943-023-01976-4

